# Editorial Comment from Dr Hirayama and Dr Iguchi to Cranial nerve palsy caused by metastasis to the skull base in patients with castration‐resistant prostate cancer: Three case reports

**DOI:** 10.1002/iju5.12265

**Published:** 2021-02-19

**Authors:** Yukiyoshi Hirayama, Taro Iguchi

**Affiliations:** ^1^ Department of Urology Osaka City University Graduate School of Medicine Osaka Japan

The skull is a common site of metastases from systemic cancers. Prostate cancer (PCa) is reported to account for 38.5% cases of skull base metastasis in a literature review of 279 patients of skull base metastasis, whereas cranial nerve palsies caused by metastatic PCa are relatively uncommon.^1^ Cranial nerve palsies appear when the neural‐foramina are compromised by tumor growth and the pattern of palsy depends on the location of metastasis.

The current article by Yasumizu *et al*. reported three cases of skull base metastasis of castration‐resistant prostate cancer (CRPC).^2^ Skull base metastases were found symptomatically after the resistance to docetaxel treatment. Although palliative external beam radiation therapy (EBRT) was attempted to the skull base metastases they all died within 3 months after the onset of symptoms. Consistent with these cases, previous studies reported that the prognosis of skull base metastasis is poor because they usually occur late in the course of progressive disease.^3^, ^4^ Radiotherapy is generally the standard treatment to palliate symptoms, however not effective to improve the poor prognosis.^4^

In all three cases, docetaxel was selected after they progressed to CRPC and the prostate‐specific antigen levels were elevated to 89.6, 209.9, and 423.9 ng/mL, respectively, at the time of diagnosis of skull base metastasis. Current therapeutic options including enzalutamide, abiraterone, cabazitaxel, and radium‐223 were not used as long as described in the article, which might because these cases were treated before the era of new agents to CRPC. It could be an option to use the novel therapeutics after docetaxel treatment to improve the outcome. Concomitant palliative EBRT is also eligible when skull base metastasis appeared.

Routine head screening to examine skull base metastasis is not always necessary in patients with metastatic CRPC; however, early diagnosis and treatments are crucial to improve symptoms and prognosis. It is important that urologists realize the possibilities of skull base metastasis in progressive metastatic CRPC. To this end, the current case report will be beneficial in clinics.

## Conflict of interest

Taro Iguchi has obtained research funding from Bayer and Astellas, has served as a consultant or advisor from Bayer and Astellas, has participated in the speakers’ bureau for Bayer, Janssen, Sanofi, and Astellas.
